# Timely assessment of 5‐year relative survival of esophageal cancer patients in Taizhou, Zhejiang province

**DOI:** 10.1002/mco2.297

**Published:** 2023-06-09

**Authors:** Min Zhang, Yongran Cheng, Xiyi Jiang, Liangyou Wang, Bicheng Chen, Tianhui Chen, Jinfei Chen

**Affiliations:** ^1^ School of Public Health Hangzhou Medical College Hangzhou China; ^2^ Department of Cancer Prevention, Zhejiang Cancer Hospital, Hangzhou Institute of Medicine (HIM) Chinese Academy of Sciences Hangzhou China; ^3^ Department of Non‐Communicable Chronic Disease Control and Prevention Taizhou Center for Disease Control and Prevention Taizhou China; ^4^ Department of Oncology The First Affiliated Hospital of Wenzhou Medical University Wenzhou China

Dear Editor,

Regarding the burden of esophageal cancer worldwide in 2020, 604,000 new cases and 544,000 new deaths were estimated according to the latest data on GLOBOCAN. The standardized incidence and mortality of esophageal cancer in China are 2.1 times higher compared to the global average. The 5‐year relative survival is an important and comprehensive indicator that reflects the overall effectiveness of cancer screening and treatment. Period analysis is a method providing timely and accurate assessment of 5‐year relative survival because it does not need 5‐year follow‐up data from cancer registries.^1^ While period analysis has widely been used in Western populations, it has rarely been used in China but was for the first time systematically confirmed in China by our group.^2^ Therefore, in this study we aimed at providing the latest data on 5‐year relative survival for patients with esophageal cancer in Taizhou, Zhejiang province, China, using period analysis.

Taizhou City accounts for approximately 10% of the population of Zhejiang Province, China. Actually, we had full access to data from all nine population‐based cancer registries from Taizhou City, Zhejiang Province, China. Data on esophageal cancer patients diagnosed during 2004−2018 and followed up till the end of 2018 were retrieved. Regarding the data quality control, we used the criteria that the proportion of death certificate only (DCO) shall be less than 13%, which indicates good quality of cancer registries as recommended by Brenner.^1^ Thereby, five cancer registries with DCO cases higher than 13% were excluded after the aforementioned quality control. Eventually we included esophageal cancer patients diagnosed during 2004−2018 from four cancer registries (Luqiao, Yuhuan, Xianju, and Wenling) that demonstrated high‐quality data. For detailed information on the methodology please refer to the aforementioned paper from our group.^2^ In accordance with the International Classification of Diseases, tenth revision (ICD‐10), we used the code C15 to classify cases of esophageal cancer. The flow chart of patient selection was shown in Figure 1A. Eventually, overall 3218 patients with esophageal cancer (2249 men and 969 women) were enrolled, including 713 cases during 2004−2008, 1191 cases during 2009−2013, and 1314 cases during 2014−2018.

**FIGURE 1:**
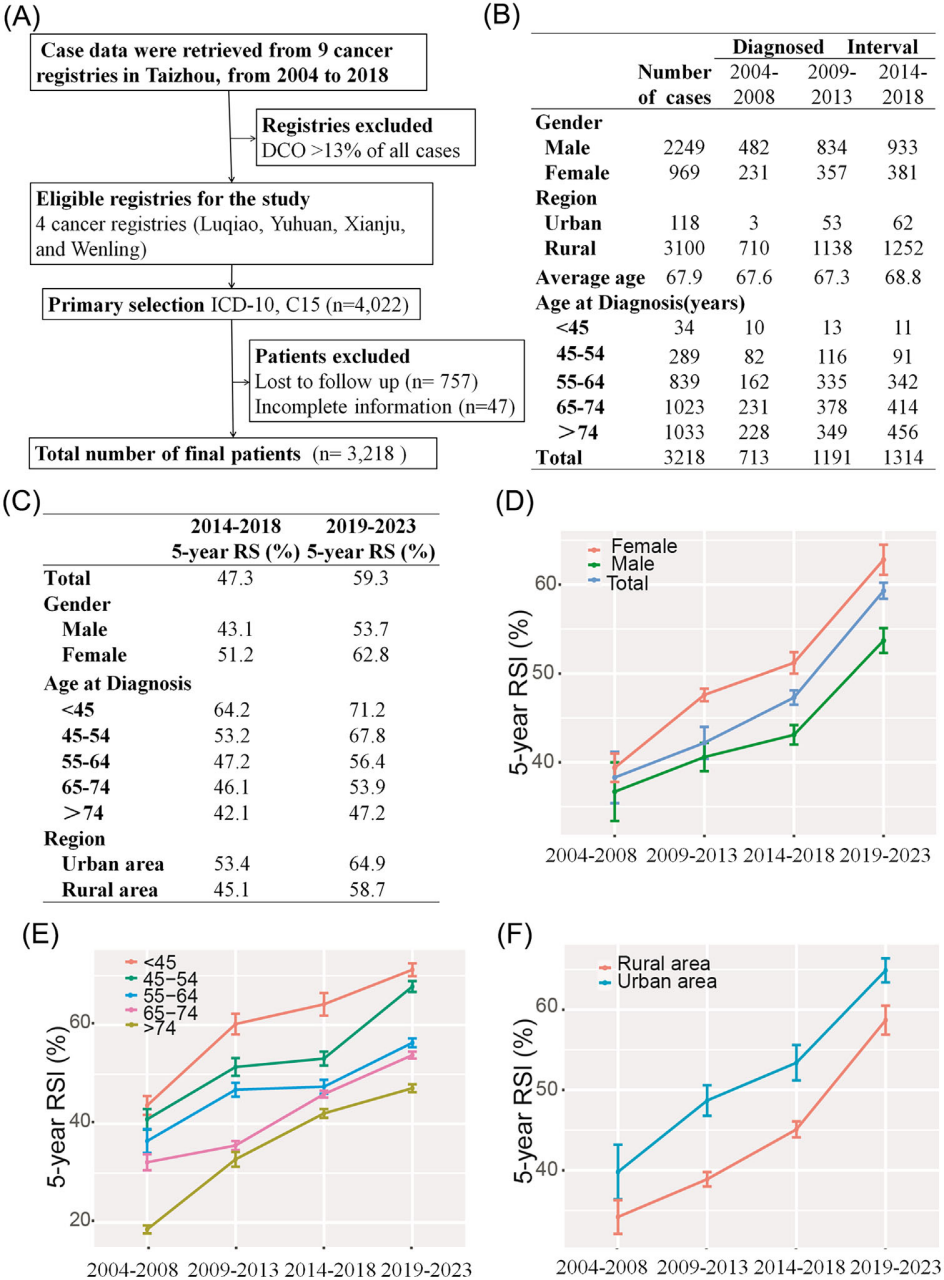
Five‐year relative survival of esophageal cancer patients from Taizhou, Zhejiang Province, China. (A) The flow chart of patients selection. (B) Basic characteristics of esophageal cancer patients during 2004−2018. (C) Five‐year relative survival for esophageal cancer patients during 2014−2018 and 2019−2023. (D) Five‐year relative survival of esophageal cancer patients from Taizhou, Zhejiang province, China during 2004−2023 for overall and the stratification by gender. (E) Five‐year relative survival of esophageal cancer patients from Taizhou, Zhejiang province, China during 2004−2023 for age at diagnosis. (F) Five‐year relative survival of esophageal cancer patients from Taizhou, Zhejiang province, China during 2004−2023 for regions.

The distribution of each period by age at diagnosis, gender and region was presented in Figure 1B. The mean age of diagnosis was 67.9 years old. Overall, only 1% patients were diagnosed before age at 45 years, while more than 60% patients were diagnosed at age 55−74 years old. In addition, the number of patients living in rural and urban areas was 3100 and 118, respectively.

To calculate the 5‐year relative survival of patients with esophageal cancer, we divided observed survival by expected survival. We used the Ederer II method to derive the expected survival from the population life tables of the aforementioned four cancer registries.^2^ We used period analysis to calculate 5‐year relative survival during 2004−2008, 2009−2013, and 2014−2018, respectively (please refer to the methodology paper from our group for details.^2^). Further survival estimates stratified by age at diagnosis, sex, and regions were also conducted. Moreover, we used model‐based period analysis to calculate 5‐year relative survival during the upcoming period 2019−2023 based on a generalized linear model and three continuous survival data (Schematic diagram is show in Table S1, while for details on the methodology for model‐based period analysis, please refer to our paper on colorectal cancer^3^).

This study provided the latest (during 2014−2018) data on 5‐year relative survival for patients with esophageal cancer from Taizhou, Zhejiang province, China, reaching 47.3%. While men had lower 5‐year relative survival compared to women (43.1% vs. 51.2%), rural areas had lower survival compared to urban areas (45.1% vs. 53.4%). Additionally, we also found an obvious age gradient, decreasing from 64.2% for patients younger than 45 years at diagnosis to 42.1% for those older than 74 years (Figure 1C). Using the model‐based period analysis, we projected that the overall 5‐year relative survival for the forthcoming period 2019−2023 reached 59.3% (53.7% for men and 62.8% for women), based on three 5‐year survival data (2004–2008, 2009−2013, and 2014−2018) (Table S1) (Figure 1C).

Our finding of 47.3% for overall during 2014−2018 is higher compared to the report of 30.3% during 2012−2015 using data from 17 cancer registries in China.^4^ However, our finding is reasonable based on the below reasons. Firstly, the aforementioned 30.3% during 2012−2015 was actually predicted. The 5‐year relative survival was estimated based on data from the aforementioned only 17 cancer registries. This was possible because the patients from the aforementioned 17 cancer registries were diagnosed by the end of 2013 and followed until the end of 2015. Therefore, the latest available data pertained to 2013 for patients with esophageal cancer (also for other cancer types) and any data beyond that year can only be predicted. Actually, survival data during 2003−2005 and 2006−2008 was calculated by cohort approach,^4^ which must be under‐estimated as confirmed by our group.^2^ Therefore, the projection during 2012−2015 must also be under‐estimated by using three continuous survival data during 2003−2005, 2006−2008, and 2009−2011. Secondly, survival of esophageal cancer patients could have improved over recent years along with more programs on early detection and screening for esophageal cancer patients, medical advances, and improved treatment. Thirdly, our data were from eastern China covering developed areas, while the data from 17 cancer registries covered less developed areas.^5^ Taken together, survival estimates on esophageal cancer patients using period approach are plausibly higher due to our data sources from developed regions with up‐to‐date data, advances on medical treatment, and more access to medical insurance.

We found the overall 5‐year relative survival has gradually increased during 2004−2018 and also during upcoming 2019−2023 (Figure 1D–F). This upward trend was consistently observed in patients of all ages, gender, and regions, implying a constant improvement in 5‐year relative survival for esophageal cancer patients from Taizhou, China. The increasing number of programs on early detection and screening for esophageal cancer, in combination of timely diagnosis and timely treatment as well as improved coverage of health insurance, could be the major reasons.

Our study has several limitations. Firstly, we could not provide stratified data by histology, stage, and treatment for the esophageal cancer patients as this kind of detailed information is commonly not available in population‐based cancer registries. Secondly, because of data availability for the current study, we only reported 5‐year relative survival of patients diagnosed with esophageal cancer for Taizhou, a city of Zhejiang province. Thereby, we are planing to provide Zhejiang provincial data in the near future.

In summary, our team provided most up‐to‐date estimates on 5‐year relative survival for patients diagnosed with esophageal cancer in Taizhou, Zhejiang province, China, using a period approach. We found that the overall 5‐year relative survival reached 47.3% during 2014−2018 and projected to reach 59.3% during the upcoming 2019−2023 period. Additionally, survival data during 2004−2018 has consistently increased for the overall and the stratification by gender, regions, and age at diagnosis. Our timely and precise survival data provide an overall evaluation on programs for early screening of esophageal cancer patients in Taizhou, eastern China, which will be beneficial to the forementioned region and neighboring areas to formulate more reasonable policy on the prevention and control of esophageal cancer.

## AUTHOR CONTRIBUTIONS

Tianhui Chen was responsible for the study concept and design. Jinfei Chen, Tianhui Chen, and Min Zhang obtained fundings. Liangyou Wang and Tianhui Chen acquired data. Yongran Cheng and Xiyi Jiang analyzed and interpreted data. Min Zhang, Xiyi Jiang, Yongran Cheng, Jinfei Chen, and Tianhui Chen drafted the manuscript, and all authors revised it for important intellectual content. All authors have read and approved the final manuscript.

## CONFLICT OF INTEREST STATEMENT

The authors declare that there is no conflict of interest that could be perceived as prejudicing the impartiality of the research reported.

## FUNDING INFORMATION

National Key Research‐Development Program of China, Grant Numbers: 2021YFC2500400 and 2021YFC2500401; Start‐up fund for the recruited talents of First Affiliated Hospital of Wenzhou Medical University, Grant Number: 2021QD025; Special project of Hangzhou Medical College, Grant Number: YS2021013.

## ETHICS STATEMENT

The data use for this study was approval by the Institutional Review Board of Zhejiang Cancer Hospital, China (IRB‐2023‐324).

## Supporting information

Supporting Information

## Data Availability

The raw data will be available for reasonable request to the corresponding authors.
