# Supplementary material for: Timely assessment of 5‐year relative survival of esophageal cancer patients in Taizhou, Zhejiang province
Source: MedComm (2020). 2023 Jun 9;4(3):e297. doi: 10.1002/mco2.297 (PMC10256990; doi:10.1002/mco2.297)
Supplement: Supplementary file 1 — Supporting Information [file MCO2-4-e297-s001.docx]

**Supplementary information**

**Timely assessment and prediction of 5-year relative survival of esophageal cancer patients from Taizhou, Zhejiang province, China**

Min Zhang^1,#,*^, Yongran Cheng^1,#^, Xiyi Jiang^1,#^, Liangyou Wang^3^, Bicheng Chen^4^, Tianhui Chen^2,*^, Jinfei Chen^4,*^

1. School of Public Health, Hangzhou Medical College, Hangzhou, China;

2. Department of Cancer Prevention, Zhejiang Cancer Hospital, Hangzhou Institute of Medicine (HIM), Chinese Academy of Sciences, Hangzhou 310022, China;

3. Department of Non-communicable Chronic Disease Control and Prevention, Taizhou Center for Disease Control and Prevention, Taizhou 318000, China;

4. The First Affiliated Hospital of Wenzhou Medical University, Wenzhou, 325000, China.

^#^These authors contributed equally to this paper.

**^*^Correspondence to**:

Prof. Tianhui Chen, Department of Cancer Prevention, Zhejiang Cancer Hospital, Hangzhou Institute of Medicine (HIM), Chinese Academy of Sciences, Hangzhou 310022, China; Email: [chenth@zjcc.org.cn](mailto:chenth@zjcc.org.cn)

Dr. Min Zhang, School of Public Health, Hangzhou Medical College, Hangzhou, China; Email: [zhangm@zjams.com.cn;](mailto:zhangm@zjams.com.cn;)

Prof. Jinfei Chen, Department of Oncology, First Affiliated Hospital of Wenzhou Medical University, Wenzhou 32500, China, Email: [jinfeichen@sohu.com](mailto:chenbicheng@hotmail.com)

**MATERIALS AND METHODS**

**Data source**

Taizhou City, with a population of 6.6 million, accounts for approximately 10% of the population of Zhejiang Province and is located on the east of Zhejiang Province. The Zhejiang Provincial Chronic Disease Surveillance System was established in 2001 as a platform to monitor the incidence and mortality of chronic diseases (including cancer) among the residents of Zhejiang Province. Case data were retrieved from 9 cancer registries in Taizhou, from 2004 to 2018. It is acceptable to evaluate the quality of the data with the proportion of death certificate only (DCO) cases less than 13% of all cancer cases. Therefore, data from the four cancer registries (Luqiao, Yuhuan, Xianju, and Wenling) were included for further analysis. As of December 31, 2018, the follow-up investigation of the life condition has been completed.

According to the tenth edition of the International Classification of Diseases (ICD-10), the cases coded C15 were identified as esophageal cancer patients. Therefore, a total of 4,022 esophageal cancer patients were initially identified, and among them, 290 were lost to followup, 47 were unknown cases, and 467 were missing at the last follow-up, which were eventually excluded. In the end, a total of 3,218 patients during 2004–2018 were included for further analysis.

Definitely, the 5-year RS estimate for patients with esophageal cancer was calculated as the ratio of observed survival in the group of esophageal cancer patients to the expected survival in a comparable group in the general population. Using the Ederer II method, according to the life tables of four cities (Luqiao, Wenling, Xianju and Yuhuan) with Taizhou population, the expected survival rate was obtained by stratification by sex, age, region and calendar year.

Period analysis calculated 5-year RS during 2014-2018. According to Brenner’s study, the research cases were divided into patients newly diagnosed in the period of interest and patients diagnosed before the period of interest but still alive in the period of interest.^8^ The research patients included patients who were newly diagnosed in 2014-2018 and who were diagnosed in 2009-2013 and still survived in 2014-2018. The calculation of period analysis was based on our previous study.^9^ First, process the data and edit it into a life table. Then, calculated 1-year RS ( *Si* ) at year *i* of follow-up as follows:

$$S_{i}\text{=1-}\frac{\text{d}_{i}}{n_{i}-c_{i}/2}$$

In this formula, $n_{i}$represents the population at the start of year i of follow-up, $d_{i}$ represents the number of deaths at the end of *i* year, and $c_{i}$ represents the number of censored data in *i* year. The observed survival $\overline{S_{k}}$ of k-year was got by multiplying the k-year conditional one-year survival rate as follows: $\overline{S_{k}}\text{=}\prod_{i=1}^{k} S_{i}$

RS (*Ri*)was calculated as follows:$R_{i}=\frac{\overline{S_{k}}}{S_{k}^{*}}$

When calculating 5-year RS, k was 5. $\overline{S_{k}}$ represents observed survival, and$S_{k}^{*}$ represents the expected survival.

This linear model, called model-based period analysis was used to predict the 5-year survival of colorectal cancer patients from 2019 to 2023 and was based on period analysis and made full use of the cancer registry data before December 31, 2018. First, the confirmed cases in 2004-2008, 2009-2013 and 2014-2018 were included according to the principles of the period analysis method (Table1). Then, calculated the number of exposures and deaths in each year, and calculated the conditional 1-year survival in each year; Finally, the conditional 1-year survival of each year was used as the dependent variable to fit the regression model (Poisson regression or binomial regression) with the follow-up period and year as independent variables.

The conditional 1-year survival r at the j period of follow-up in i year after diagnosis is expressed as: r_ij_= exp (-exp(α_ⅰ_ +j×β ))

Where j represented the follow-up period, j=0 represents 2004-2008, j=1 represents 2009-2013, and j=2 represents 2014-2018. I represents the number of years of follow-up in each period. For example, from 2004 to 2008, i= 1,2004, i=2, 2005, and so on.

The cumulative 5-year relative survival of follow-up in the j period is expressed as:

Ｒj = Πr_ij_ = Πexp (-exp(α_ⅰ_ +j×β ))

All statistical analyses were implemented with the "periodR" software package of R version 3.13 (R Foundation for Statistical Computing, Vienna, Austria).

Table S1 Schematic diagram of model-based cycle

| **Diagnostic period** | **Follow-up period** | | | |
| --- | --- | --- | --- | --- |
|  | **2004-2008** | **2009-2013** | **2014-2018** | **2019-2023** |
| **1999-2003** |  |  |  |  |
| **2004-2008** |  |  |  |  |
| **2009-2013** |  |  |  |  |
| **2014-2018** |  |  |  |  |
| **2019-2023** |  |  |  |  |

represented cases confirmed before the period of interest but still surviving during the period of interest

represented newly confirmed cases during the period of interest
